# Meeting physicians’ needs: a bottom-up approach for improving the implementation of medical knowledge into practice

**DOI:** 10.1186/s12961-016-0120-5

**Published:** 2016-07-18

**Authors:** Carla Vaucher, Emilie Bovet, Theresa Bengough, Vincent Pidoux, Michèle Grossen, Francesco Panese, Bernard Burnand

**Affiliations:** Healthcare Evaluation Unit, Institute of Social & Preventive Medicine (IUMSP), Lausanne University Hospital, Biopôle 2, Rte de la Corniche 10, CH-1010 Lausanne, Switzerland; Austrian Federal Institute of Healthcare (ÖBIG), 1010 Vienna, Austria; Institute of Psychology, University of Lausanne, Lausanne, Switzerland; University Institute of the History of Medicine and Public Health, Lausanne University Hospital, Lausanne, Switzerland; Liaison Psychiatry, Service of Psychiatry, Lausanne University Hospital, Lausanne, Switzerland; University of Health Sciences (HESAV), Lausanne, Switzerland; Cochrane Switzerland, Institute of Social & Preventive Medicine (IUMSP), Lausanne University Hospital, Lausanne, Switzerland; STS Lab, University of Lausanne, Lausanne, Switzerland

**Keywords:** Knowledge translation and implementation, Improvements, Interviews, Focus group discussions, Online survey, Qualitative methods, Qualitative analysis, Physicians

## Abstract

**Background:**

Multiple barriers to knowledge translation in medicine have been identified (ranging from information overload to abstraction of models), leading to important implementation gaps. This study aimed at assessing the suggestions of practicing physicians for possible improvements of knowledge translation (KT) effectiveness into clinical practice.

**Methods:**

We used a mixed methods design. French- German- and Italian-speaking general practitioners, psychiatrists, orthopaedic surgeons, cardiologists, and diabetologists practicing in Switzerland were interrogated through semi-structured interviews, focus group discussions, and an online survey.

**Results:**

A total of 985 physicians from three regions of Switzerland participated in the online survey, whereas 39 participated in focus group discussions and 14 in face-to-face interviews. Physicians expressed limitations and difficulties related to KT into their daily practice. Several barriers were identified, including influence and pressure of pharmaceutical companies, non-publication of negative results, mismatch between guidelines and practice, education gaps, and insufficient collaboration between research and practice. Suggestions to overcome barriers were improving education concerning the evaluation of scientific publications, expanding applicability of guidelines, having free and easy access to independent journals, developing collaborations between research and practice, and creating tools to facilitate access to medical information.

**Conclusions:**

Our study provides suggestions for improving KT into daily medical practice, matching the views, needs and preferences of practicing physicians. Responding to suggestions for improvements brought up by physicians may lead to better knowledge translation, higher professional satisfaction, and better healthcare outcomes.

**Electronic supplementary material:**

The online version of this article (doi:10.1186/s12961-016-0120-5) contains supplementary material, which is available to authorized users.

## Background

Multiple models, studies and developments aiming at fostering the connections between scientific research findings and medical practice have been looked into in recent decades, encouraging the use of guidelines, systematic reviews of effectiveness, or validated tests and treatments in clinical practice and policymaking [1]. However, research showed that practicing physicians did not refer systematically to evidence-based medicine (EBM) studies and tools, such as clinical guidelines, in their daily practice [2, 3]. Consequently, strengthening the relationship between knowledge derived from scientific research and daily clinical practice represents an important challenge. The question of how to exchange, synthesize and integrate the medical knowledge created by researchers so that practitioners may find concrete and meaningful applications in their actual practice remains to be answered [4].

Many studies have highlighted different barriers to knowledge translation (KT) in medicine, ranging from speed of information and changes in communication technologies to a lack of access to systematic reviews and appraisal of relevance of the studies [5–7]. According to physicians, obstacles were shown to be especially related to patients’ demands, a lack of time [8], mismatches between guidelines and individual patients’ reality [9], individual preferences [10], lack of evidence appraisal and statistical skills [11], resistance to change, or misunderstandings concerning the benefits of using a new device [12]. An implementation gap in the medical field, also referred to as a ‘know-do gap’, has been well documented [13–15]. It describes the difficulty of integrating evidence into practice [4], often because of the mismatch between knowledge supply and its use in practice, or the lack of dialogue between researchers and practitioners [16–18].

Thanks to an effort to bridge the gap between what is known about potential solutions for healthcare issues and what is actually done to implement them into practice [19], some likely solutions have been put forward. They can be divided into two main groups. On the one hand, strategies seeking to (re)connect scientific evidence with clinical practice include educational materials, scientific meetings and conferences, or conversations with colleagues and local opinion leaders [20]. They may also focus on how to support physicians and other healthcare professionals facing an information overload, or carry out knowledge application into daily practice through a better understanding of its context of supply [21]. On the other hand, many suggestions seem to be technological, and concentrate on the creation of software and hardware solutions for the storage, transfer and retrieval of information in the medical setting [17, 22]. Computerized and summarized databases, web portals, intranet applications, computer-based decision supports, agent-based networks, and electronic knowledge management systems have been created to help overcome KT difficulties [13, 23, 24]. A shared goal of these technological tools is to foster an easy and timely access to a wide array of information, to support decision-making, and to accelerate KT into medical practice. Technological tools such as electronic health records are also designed to improve the safety, quality, and efficiency of care within hospitals [25], to facilitate communication between services [26], and to improve physicians’ compliance to medical guidelines [27].

However, the proliferation of KT models and technical devices could lead physicians to confusion and to an additional feeling of being overwhelmed. Indeed, implementation of new information devices may imply an additional workload for physicians when learning how to use them, or how to manage patients’ data [12]. Many KT suggestions drawn from the literature are part of a top-down process including solutions generated by small groups of knowledge providers and disseminated worldwide to a broad and undifferentiated population of health practitioners [28]. In contrast, we assume that a bottom-up approach in the field of KT is needed in order to elaborate and disseminate solutions generated by the practicing actors themselves. The use of a bottom-up approach in order to find KT solutions within the field of healthcare enables taking into account professionals’ needs and requirements. Bottom-up approaches, such as user-assisted design, could help develop local and meaningful tools for a targeted population, improving medical practice in innovative ways [16, 29, 30]. Indeed, it has been shown that new information and communication devices are more likely to be integrated into practices if they rely on pre-existing practices and representations [30]. Bottom-up approaches allow for the initiation and implementation of innovative processes and tools relying on target groups’ experience, needs and ideas, leading to concrete and user-sensitive solutions. Such an approach empowers target groups by considering them as the actual implementers of new practices and representations. On the contrary, a top-down process would base actions and recommendations to users (in our case, physicians) on governments or recognized policymakers’ opinions and decisions. Thus, a top-down process gives more control to external agents, whilst a bottom-up process gives more power and control to the users themselves.

In response to the proliferation of supposedly universal theoretical models of KT, and to the usual recommendations concerning how documented barriers should be overcome, we aimed at developing a local and concrete bottom-up assessment of successful and unsuccessful translation of research-based knowledge into daily medical practice. Assuming that practicing physicians, as knowledge users, should participate in the creation of the tools they have to use and to share in their daily practice, our general objective was to capture physicians’ difficulties in accessing information in their daily practice, in order to identify barriers to KT. More specifically, the study pursued four goals: firstly, to improve knowledge about concrete practices and habits of physicians regarding research and the use of relevant medical information in their daily practice. Secondly, to explore the sources of information considered in their clinical practice, as well as their professional interest for EBM. Thirdly, to interrogate their expectations and suggestions regarding the implementation of new scientific knowledge in their daily clinical practice. Finally, to take into account the views, practices and context of physicians practicing in Switzerland, to provide concrete suggestions for improvements of KT effectiveness into medical practice.

## Methods

### Study setting

We used a mixed methods design [31], consisting of 14 face-to-face semi-structured interviews [32, 33], five focus group discussions [34], and an online self-administrated questionnaire involving practicing physicians from the French-, Italian- and German-speaking regions of Switzerland. This mixed design was chosen so that the experiences and representations of physicians could be explored more fully in order to find out potential improvements in KT effectiveness for their daily clinical practice. Focus group discussions (FGDs) and interviews were conducted in the French- and German-speaking regions of Switzerland between September 2012 and September 2013. Self-administrated questionnaires were completed by French-, German- and Italian-speaking physicians between May and July 2014. Interview guides used for the interviews and focus group discussions contained specific questions about barriers to the application of evidence into practice and perceived possible improvements. Participants were explicitly asked to offer concrete suggestions for a better integration of scientific knowledge into daily practice. This paper focuses on data derived from (1) 14 semi-structured face-to-face interviews in which physicians suggested improvements, (2) five FGDs in which physicians suggested improvements, and (3) one open-ended question drawn from an online survey on KT in medicine.

### Participants

Participants were recruited for FGDs and interviews using a random sampling technique based on the following inclusion criteria: (1) holding a Swiss Medical Association specialty diploma for a minimum of 5 years, (2) not having a hospital affiliation, and (3) practicing in Switzerland.

The criterion stipulating that the participants should have received their diploma at least 5 years before the study was based on the hypothesis that physicians in practice for more than 5 years would have developed habits in relation to their use and representations of EBM in their practice, which could contrast with the education they received during their training, and the practices observed in the academic field and in hospitals. Further, access to online databases is facilitated in Swiss University Hospitals, which have subscriptions to a wide range of leading medical journals. Hospitals and Universities also facilitate access to laboratories, imaging techniques, and more continuous training than afforded to private practitioners. Physicians practicing in hospitals were expected to be more aware of existing journals and databases, and to have more contacts with other professionals and institutions, possibly leading to a greater inclusion of EBM practices. The reasons for choosing the aforementioned criteria were based on the hypothesis that remoteness from ‘EBM centres’ could result in a critical distance or in more difficulties in including EBM strategies into daily practice.

The majority of physicians who participated in the study worked in a medical practice, either alone or with one or two other physicians. Physicians were recruited via postal letters (contact details were retrieved from the database of the Swiss Medical Association) as well as via email, for participation in the interviews and focus group discussions, with the support of the respective specialty societies. Those participating in the online survey were contacted via email by a Swiss company specialized in social research (GFS Bern), which was responsible for the management of the survey (for instance, the company was in charge of sending reminders to participants).

Respondents for this study consisted of 1038 practicing physicians from five different specialties, including general practice, psychiatry, cardiology, diabetology and orthopaedic surgery, surveyed through three different means:

- A total of 6400 physicians were contacted to participate in an online survey; 985 physicians completed the online self-administrated questionnaire; 295 answered in German, 131 in French, and 30 in Italian; 97 were women and 354 were men.
- A total of 14 physicians (six German- and eight French-speaking) participated in individual semi-structured interviews. Schedule constraints on the physicians’ side made the recruitment phase difficult and led to fewer interviews than initially planned.
- A total of 39 physicians (14 German- and 25 French-speaking) participated in five different FGDs. Groups were comprised of five to nine participants. A lower participation rate of participants from the German-speaking region explains the imbalance of FGDs across the two regions.

### Study design

#### Interviews

Face-to-face, semi-structured interviews were held in French and German, using an interview guide based on a literature review developed by two of the authors (EB, VP).

Interviews were held by trained interviewers (EB, TB, VP), who informed the participants about the confidentiality of the data and the possibility of withdrawing from the research at any time. All interviews were audio-recorded with the participants’ consent and transcribed by the interviewers. All identification information was removed from the transcripts. All interviews but one took place at the participants’ workplaces and lasted approximately 1 hour. All physicians involved received a financial compensation for their participation in the interviews or in the FGDs, calculated on the duration of the encounter. The amount was around 160 CHF (equivalent of 160 USD) per hour for interviews and FGDs, in addition to travel expenses, according to usual rules at Lausanne University Hospital. No financial compensation was offered to participants in the online survey.

Participants were given information about the methodological steps of the study and its interdisciplinary nature. They were also informed about the four objectives of the study. The interviews all began with a presentation of the physician’s professional career and interests, and then went on with the five broad topics comprised in the interview guide. The first one was the sources of information they used, followed by evaluation of relevance and utility of scientific information in medicine, translation of scientific knowledge into daily practice, satisfaction and opinions in relation to EBM, and research progress over the last 30 years.

#### FGDs

The exploratory interviews served as a basis to develop the discussion guide for the focus group discussion moderators’ exchange with the practicing physicians. FGDs [35] were considered relevant to grasp the perceptions of the physicians’ on KT in their everyday practice, and the possible weaknesses and successes of KT. The group discussions focused on topics that emerged from the face-to-face interviews: ways and means used to keep up to date with relevant information, perceived role of physicians within medical science, potential barriers to KT, and possible suggestions of improvement for effective KT.

Five FGDs were held; two in the German- and three in the French-speaking part of Switzerland, involving a total of 39 physicians (Table 1). Each focus group involved five to nine participants and lasted between 90 and 120 minutes, in alignment with qualitative methods recommendations concerning the management of FGDs [34, 36, 37].

**Table 1:** Participants in interviews and focus group discussions, by specialty

Number of	Orthopaedists	Psychiatrists	Cardiologists	Diabetologists	General practitioners
Participants in interviews	3	2	3	2	4
Participants in focus group discussions	0	14	0	0	25
Total	3	16	3	2	29

Discussions were held either in our premises or in an external conference room. All FGDs were audio-recorded and transcribed verbatim. FGDs were conducted by trained moderators (EB, TB, VP), assisted by a co-moderator taking additional field notes in order to facilitate further transcriptions of the audio-recorded discussions. Shortly before the FGDs took place, the participants received an email containing the list of participants as well as a short summary of the topics to be discussed.

#### Online survey

The analysis of the data from the interviews and FGDs led to the creation of an online survey. The survey comprised 32 questions, including six socio-demographic items (age, sex, specialty, current employment status, years of practice, and location of practice). Among the 26 core questions, 20 were close-ended questions, four were semi-open, and two were open-ended. In this article, we focus on the last open-ended question, question 26, which asked the respondents to suggest concrete improvements for practical implementation of knowledge derived from scientific research. The exact wording of question 26 was: ‘What would your solutions be in order to concretely improve the practical implementation of research-based knowledge?’ The complete online survey results will be discussed in another publication. Qualitative results regarding the specific case of general practitioners at a Swiss level have been published by members of our research team, offering a first glance on practitioners’ perception of KT [38].

### Data analysis

Each set of data was analysed according to the method used (interviews, focus group discussions, survey), enabling an in-depth exploration of their content. All transcripts of the interviews and FGDs were reviewed by the three investigators who collected the data and two research assistants (CB, SS). In-depth qualitative content analysis [39] was undertaken, based on the grounded theory method, enabling us to identify broad content categories [40].

After getting familiar with the data, two researchers (EB, TB) identified the main themes emerging from the data and listed them into a spreadsheet. The themes were coded independently by the two researchers and then discussed together. A third researcher was consulted to settle possible disagreements in coding. A coding rulebook was established and adapted throughout the coding process. In the second phase of the coding process, a qualitative lexical data analysis software (Iramuteq^®^) was used, helping to sort out and quantify segments of the corpus, which led to defining the themes that reflected the views and opinions of the participants in the interviews and FGDs. The online survey data was not included in the Iramuteq software, because the survey comprised more close-ended, and thus quantitative, data. We therefore chose to use the software only for the qualitative analysis of the general practitioners’ interviews.

In the last step, interviews and FGDs were read through and analysed again by two other researchers (CV, MW) who had not participated in data collection, in order to have an external view of the analysis. Thematic categories were created and compared with the pre-existing codes and categories designed by the researchers previously mentioned (EB, TB) and the qualitative analysis software. FGDs and face-to-face interviews were then cross-analysed to highlight comparisons between groups of participants, for example, between specialties or linguistic regions. The excerpts included in this article were translated from German, Italian and French into English. Original excerpts can be found in Additional file 1.

Participants in the study were not involved in the coding process or thematic analysis, and they were not asked to validate limitations and suggestions presented in this text. However, they will receive a feedback on publications related to their participation to the study, as well as the online survey results, by e-mail.

### Researchers’ reflexivity

The interdisciplinary research team was of great value during the whole research process. Indeed, the collaboration of a physician, a social psychologist, science and medicine historians, anthropologists and statisticians allowed for a greatly enriched reflection process during the elaboration of the online survey, the construction of interview and focus group guides, and during the analysis of the rich quantitative and qualitative data. Further, two of the research team members (EB, VP) have been working on a KT mechanisms critical literature review (under review), which certainly had an impact on the developmental stage of the research.

Working for the Institute of Social & Preventive Medicine at the time of the data collection certainly also had an impact on the participants’ expectations of what would be provided to them. Indeed, participants may have considered us as potential ‘appraisers’ of their professional practices on the one hand, and/or as ‘solution providers’ on the other. Those issues were actually explicitly discussed during some of the encounters.

## Results

Although the great majority of physicians confirmed their general knowledge and use of EBM tools within their daily medical practice through the online survey, many of them expressed some difficulties, critiques and suggestions for KT improvements during the interviews and FGDs, and answered the online survey question providing concrete solutions for everyday practice.

Among the 985 (15.4%) physicians who participated in the online survey, 456 (46.3%) answered the question about possible improvements (question 26); 295 answered in German, 131 in French, and 30 in Italian; 97 were women and 354 were men. The majority of respondents to this question were family physicians (n = 297), followed by psychiatrists (n = 69), orthopaedic surgeons (n = 59), cardiologists (n = 23) and diabetologists (n = 8) (Table 2).

**Table 2:** Respondents to online survey question number 26, by specialty

	Orthopaedists	Psychiatrists	Cardiologists	Diabetologists	General practitioners
Number of respondents to question 26	59	69	23	8	297
Proportion of respondents, %	35	42	48	47	51
Total participants to online survey	168	165	48	17	587

### Barriers to KT into daily practice

Analysis of the individual interviews, FGDs and online surveys revealed several perceived barriers to KT into daily practice. The topic of barriers to KT emerged mostly during the face-to-face encounters with physicians, while concrete suggestions for improvements were primarily derived from answers to the online survey question.

Five main perceived barriers were described by the physicians when referring to KT in their daily practice: (1) influence of pharmaceutical firms; (2) publication biases; (3) mismatch between clinical guidelines and practice; (4) education gaps; and (5) lack of collaboration between research and practice. General suggestions for improvements matched those five barriers in KT.

#### Barrier 1: Pharmaceutical companies

The issue of influence and pressures from pharmaceutical companies was as frequently raised within interviews and FGDs as in the answers to the online survey. Almost all physicians stressed that the influence of pharmaceutical companies on publications, education and knowledge supply was a problem. More specifically, physicians were quite aware of the interferences between pharmaceutical firms and clinical studies. They expressed doubts, mistrust and scepticism about scientific results due to biases related to pharmaceutical companies. Several issues were largely discussed, including the presence of advertisements for drugs in scientific journals, suspicions of complicity between pharmaceutical firms and the emergence of new diagnoses, the fact that some congresses are sponsored by pharmaceutical firms, or that some medical studies direct the discussion towards the issue of insurances reimbursement.

Excerpt 1: Interview with German-speaking cardiologist:

“*And it simply doesn’t work without the pharmaceutical industry. It doesn’t work, we need the money. Who is paying it otherwise? The Swiss National Science Foundation is more about basic research and such. I know that we need the money*.”

#### Barrier 2: Non-publication of negative results

In relation to the preceding barrier, several physicians raised the issue of the non-publication of negative results, which was considered as another bias that weakened the quality of scientific research. This barrier was not addressed as much within interviews and FGDs as within the survey answers, where it appeared more often. The physicians criticized the fact that positive results were preferentially published by scientific journals, especially when it came to randomized controlled studies. Biases related to publication pressures, both on the researchers and the journals’ sides, were seen as impairing the progression of science in general.

Excerpt 2: Online survey answer by Italian-speaking general practitioner:

“*Research should be less dependent on the pharmaceutical industry. Studies containing negative results should also be published*.”

#### Barrier 3: Mismatch between guidelines and practice

Although the online survey revealed an extensive use of guidelines by practicing physicians, especially by cardiologists and orthopaedic surgeons, a topic that was highly discussed during interviews and FGDs was the mismatch between guidelines and daily practice. This barrier was the most extensively and frequently discussed topic during interviews and FGDs, and also emerged several times in the survey answers. However, results obtained through the FGDs and interviews were slightly nuanced by the answers to the online survey, in which physicians expressed a widespread use of clinical guidelines, while also expressing their wish for them to be more accessible and improved.

Participating physicians considered guidelines as inconsistent with usual situations met with patients. Patients were considered as irreducible to the guidelines’ pre-established categories due to the specificity of each clinical situation. This topic was especially raised by psychiatrists and family physicians, who reported that they were facing situations that, for the most part, did not fit clinical recommendations. Consequently, they considered guidelines as inappropriate and non-representative tools for their daily practice. The physicians also considered guidelines to be behind the advancements of medical practice. One of the paradoxes they raised was that pharmaceutical firms were sometimes found to be more up-to-date than academics and practitioners when it comes to new treatments. The physicians viewed guidelines as unstable, unreliable and non-pragmatic, and sometimes as an obstacle to independence of thought.

Excerpt 3: FGD with French-speaking general practitioners:

“*In my opinion, guidelines are, in fact, tools, and I find, more and more, that these tools are very hardly appropriate to our work. They are based on measures, which, in fact, change all the time* […] *actually we have tools that are very badly calibrated to what we do, and sometimes not even very reliable.*”

#### Barrier 4: Training gaps

Education was a topic addressed by many physicians during the interviews, FGDs, and in the online survey questions. The physicians spotted several gaps in medical education, including the disappearance of the obligation to get an MD in order to obtain a Swiss Medical Federation specialty diploma, the lack of time and energy required to update one’s knowledge and participate in scientific events, and a lack of methodological competences to assess the quality of scientific research. These perceived gaps in education were considered as resulting in poor skills in the understanding of scientific publications. Psychiatrists and general practitioners expressed doubts regarding their methodological competences when it came to evaluating the scientific relevance of a study. The Swiss system of continuing medical education was also widely criticized in the face-to-face interviews. According to the surveyed physicians, the system promoted ‘scientific tourism’, and physicians tended to participate in congresses in order to collect certificates of attendance rather than to gain greater knowledge.

Excerpt 4: Interview with German-speaking general practitioner:

“*I don’t have the background to interpret the statistical part of a study. Primarily this is due to the fact that I don’t carry out research myself. Secondly, because we haven’t been trained in this area. I have to say that during university classes, reading statistics in a sense, applying, you know, having some formulas…*”

#### Barrier 5: the know-do gap

Another topic of concern raised by the physicians was the absence of collaboration between research and practice. This barrier appeared less under the form of a definitive obstacle than as a suggestion for more collaboration, which will be discussed further below. Physicians reported to experience a lack of partnerships between their daily practice and scientific research, and considered that academic institutions did not benefit from clinical experience and, conversely, that clinicians did not benefit enough from knowledge developed in academic institutions. They also reported a growing gap between scientific publications, official decision-making tools, and daily clinical situations. Finally, according to them, the patients’ wishes and requests did not often match clinical recommendations or raw data found in the literature. Excerpts 5 and 6 are representative of physicians’ answers:

Excerpt 5: Interview with German-speaking psychiatrist:

“*Maybe research is too specific, too specialized… or maybe it just lost its relation to practice a little bit*.”

Excerpt 6: Online survey answer by French-speaking psychiatrist:

“*Improved synergy between research, practice and teaching. Integration and value of practice and clinical experience at university level* [research and teaching]”.

### Suggestions for improvements

The respondents’ suggestions were classified either as general or as concrete. General suggestions comprised ideas that were considered as possible improvements for KT from the research team’s perspective, but that were not accompanied by concrete ideas concerning how to implement them. On the contrary, concrete suggestions gather physicians’ suggestions for actual tools that could improve daily application of knowledge derived from research and daily information-sharing. General suggestions were mainly expressed in the FGDs and interviews, while concrete solutions were mainly brought up in the online survey question.

### General suggestions for improvements

The five general suggestions presented below mirror the five barriers reported above.

#### General suggestion 1: Independence from pharmaceutical firms

Regarding the influence of pharmaceutical companies on knowledge and publications, the physicians insisted on the need to keep a critical eye when confronted with scientific publications, and to be aware of possible biases when judging the methodological quality of a study. They suggested developing web-based platforms and journals, and creating guidelines that would be independent from any pharmaceutical influence. They expressed the wish to have the possibility of attending congresses and presentations without the interference of potential conflicts of interests. This suggestion was mostly given as an answer to survey question 26 and discussed more implicitly during interviews and FGDs.

Excerpt 7: Online survey answer by German-speaking general practitioner:

“*Guideline authors should be independent from stakeholders. Then one could rather trust them. Pharmaceutical research should be carried out by national institutions. One should remove pharmaceutical, apparatus, hotel and other industries from medical education. Guidelines should not be sponsored by the industry primarily*”.

#### General suggestion 2: Transparency

The physicians considered that scientific publications should show full transparency with regards to all obtained results. They expressed the need to improve research and scientificity by upgrading scientific integrity and reliability. They also wished for more local research, especially in the field of general practice. Several online survey answers comprised a suggestion for ‘improving research’ in general, without further specifications. This suggestion was also more frequently expressed via the online survey than directly during face-to-face encounters.

Excerpt 8: Online survey answer by French-speaking general practitioner:

“*A better selection of studies by scientific journals, in order to be less suspicious when facing the results*.”

#### General suggestion 3: Discuss and adapt guidelines

Suggestions were made to improve accessibility to guidelines in terms of price, speed and ease, to keep them concise and up-to-date, and to have access to more local guidelines, elaborated by Swiss experts. By concise, physicians meant that they needed short and ready-to-use information, such as e-mails containing one line for each new research finding that may impact their field of practice. In order to remain up-to-date, the physicians suggested the creation of a web-alert when new guidelines for a specialty were developed. They also suggested discussing guidelines among practitioners to examine the difficulties regarding their application and usefulness in their practice, and possibly including patients’ associations, as consumers, when reflecting on guidelines. Discussing daily individual situations of clinical practice with colleagues was often reported to be more helpful than consulting guidelines.

Another result related to clinical guidelines was that physicians did not consider them as impenetrable obligations. Instead, they suggested to adapt them slightly to each specific situation met in their daily practice. The physicians mentioned the importance of knowing guidelines without, however, bestowing them with an absolute value.

Suggestions related to discussing and adapting guidelines were mostly raised during face-to-face encounters, and especially during focus group discussions, where the physicians exposed their views on the constant evolution of guidelines and their mismatch with practice.

Excerpt 9: Interview with French-speaking diabetologist:

“*I believe that both in medical practice, like in music, one needs to know the rules very well to allow oneself to take a certain distance without causing any harm*.”

#### General suggestion 4: Improving methodological competences

General suggestions for education indicated the need to improve methodological skills both during academic and continuing education, especially those needed for the interpretation of scientific literature and the creation and development of collaborative networks. One general practitioner also suggested periodic random medical examinations (once every 5 years) to replace the collecting of certificates of attendance at various scientific events. These suggestions were mainly expressed in the FGDs and interviews, in which several physicians expressed difficulties in judging the scientific relevance of a study or evaluating the quality of statistical results presented in scientific publications.

Excerpt 10: Interview with German-speaking cardiologist:

“*The one thing is understanding all these technical terms, the protocol… I have therefore participated in a short statistical training. To really grasp better what it means, the relative risk and such… whether it really is as significant as it looks …*”

#### General suggestion 5: Collaboration

In their answers to the question regarding collaboration between researchers and practitioners, physicians insisted on the importance of creating networks and sharing experiences between academic research and clinical application, between hospitals and private practice, between general practitioners and specialists, and between colleagues within the same specialties. Their underlying idea was to involve all stakeholders in order to improve KT and application. This would help create more pragmatic solutions focused on daily practice. Several physicians emphasized the importance of valuing practitioners’ experiences and practice. The answers to question 26 of the online survey also included general suggestions for more proximity between academics and actual practitioners. Participants considered that every physician should participate in research in order to familiarize themselves with its methods. Finally, they also suggested that the physicians’ practical experience should be valued and trusted.

Excerpt 11: Interview with French-speaking orthopaedic surgeon:

“*What I have always hoped for* […] *is to involve people who are in private practice in public hospitals, to prevent them being totally isolated from what happens in academic centres. It is to build a network* […] *it is more interactive, and more motivating for people to somewhat change their daily routine a little.*”

### Concrete suggestions for improvements

The physicians mentioned several concrete suggestions for improvements of KT into daily practice in the interviews, FGDs and the online survey. Concrete suggestions mostly concentrated on information-sharing tools, type of information needed, and data accessibility.

#### Concrete suggestion 1: “Pre-digested” information

The first line of suggestions concerned the type of data needed. Physicians were very clear about the form and substance of information they needed to support their daily practice. General practitioners were the most directly affected by information overload from a wide variety of sources, characterized as an ‘information jungle’ by one of the focus group participants; these physicians especially expressed the need for synthetic, summarized, unified, clear, and up-to-date information, in relation to the variety of topics with which they have to deal. They reported a need for pragmatic information that would be applicable to the unique situations they face daily. This precision enables us to make a difference between knowledge providers and knowledge users. As knowledge users, general practitioners expressed a need for applicable knowledge that would correspond to their daily situations and would be adapted to each specialty. Several participants across different specialties were also willing to receive regular ‘pre-digested’ information from recognized experts in their medical field.

To obtain concise, summarized, clear and independent information, physicians mentioned using two means, sometimes combined – medical experts and technological tools. Indeed, a first group of suggestions outlined the idea of bringing together experts from each medical field with the aim of reviewing the latest relevant discoveries for clinical practice, and to publish an independent synthetic newsletter containing their recommendations and advice about the latest treatment developments and the latest relevant publications. A second group of suggestions, ensuing from the latter, was to develop online platforms that would collect summaries of the latest relevant scientific knowledge from each discipline, edited by experts from each medical field. According to the respondents, these web-portals could consist of one page a day including a one-line summary for each medical field, with pre-sorted, unified and simplified information. This second group of suggestions was considered as especially relevant for general practitioners, who have to take into account several medical specialties in their daily practice.

Excerpt 12: Focus Group with French-speaking general practitioners:

“*What appears to me, in general, in medicine, with respect to information overload, is that a pre-selection is missing. There is no sorting. We are overwhelmed with crashing waves of information and I think there should be a selection. Personally, for example, I dream to receive, once a day,* […] *a sheet with one synopsis* […] *something super simplified* […] *so much energy could be saved* […] *by pre-digesting the work a little* […] *a kind of real abstract for each discipline, we don’t have that, it’s a little anarchic I find*.”

#### Concrete suggestion 2: Technological tools

The second line of concrete suggestions for improvements concerned technological tools. A few physicians, especially general practitioners and psychiatrists, still preferred books, journals and direct contact with colleagues, or expressed difficulties in using computers and the Internet. Nevertheless, most participants suggested improving existing technological tools for better KT. Physicians wished for many possible improvements related to Internet tools, web-based sharing platforms or tablet applications. Their suggestions for technology improvements included using the website of a public service institution as a means of receiving notifications concerning fundamental changes in practice once a month, developing websites with permanent updates, creating web-based discussion forums involving field experts instead of theorists removed from reality, and developing an online EBM service financed by the state. The physicians also insisted on the need for the publication of local research results. Indeed, they suggested the creation of a Swiss version of the UpToDate® or similar web platforms [41, 42] related to regional concerns, an EBM website gathering Swiss recommendations, or the creation of specifically Swiss guidelines for each medical field, knowing that there is no national agency responsible for the publication of guidelines in Switzerland.

Excerpt 13: Online survey answer by Italian-speaking psychiatrist:

“*Improve scientific websites for tablets*.”

Excerpt 14: Online survey answer by Italian-speaking general practitioner:

“*Give access to a computer program with continuous updating*.”

#### Concrete suggestion 3: Quick, free and easy access to information

One last area for possible improvements pointed out by the participants concerned information accessibility. This issue was raised mostly by general practitioners and psychiatrists in the FGDs and interviews. The physicians insisted on the need for an easier access to online publications, in terms of price and convenience. They suggested providing free access to all publications and online medical platforms, not only for hospital institutions, but also for private practices, in order to improve KT into practice. Free and improved access to web-platforms and articles was also mentioned several times in the answers to online survey question 26. Physicians also suggested improving access to published research results through tablets and smartphone devices. They considered that access to medical publications and web-portals should be easier and quicker, with direct links to synthesized and pre-ordered information.

Excerpt 15: Online survey answer by Italian-speaking orthopaedic surgeon:

“*Easy and free access to journals*.”

Excerpt 16: Online survey answer by French-speaking cardiologist:

“*Broader and cheaper access to online medical journals*.”

#### Concrete suggestion 4: More time

Finally, the physicians expressed the need for paid time in their daily practice that could be devoted to a systematic reading of medical journals and keeping up-to-date with medical developments. While the online survey answers frequently showed very simple formulations containing the word ‘time’, or comments about the need for more time and less pressure for physicians, the issue of lack of time was especially raised during interviews and FGDs and in particular by general practitioners, who expressed difficulties in dealing with their schedules.

Excerpt 17: Online survey answer by Italian-speaking cardiologist:

“*More time… Less pressure*.”

The findings of this study enabled us to define five concrete recommendations to meet the needs and preferences of practicing physicians in relation to KT (Box 1).

## Discussion

The face-to-face encounters and the online survey answers by physicians from five different specialties and from three linguistic regions provided further evidence concerning barriers to KT in Switzerland. Study participants showed great awareness of EBM and KT and seemed critical towards scientific information. However, clinical guidelines, systematic reviews and scientific articles aiming at helping KT sometimes seemed to be perceived as quite inaccessible in terms of price, time, scientific credit and possibility of application to daily clinical situations. This was especially true for general practitioners and psychiatrists. In line with previous literature [8], the surveyed physicians considered that guidelines were not applicable to individual patients’ lifestyles and realities. Similarly to our findings, complex guidelines, a lack of time and resource constraints have also been identified previously as reasons for non-adherence to clinical practice guidelines [43].

Means involving direct contact with experts and colleagues, such as collaboration networks, were particularly appreciated by general practitioners and psychiatrists, which is consistent with previous literature [44, 45]. Indeed, physicians seemed to favour telephone or face-to-face contact with colleagues from the same medical field and from other specialties to acquire up-to-date and pragmatic information relevant to daily situations. Relying on a colleague or referring to a known specialist has previously been identified by the literature as a prioritized means compared to personal information searches. When confronted with an unsolvable issue, contacting someone they trust allows physicians to save a considerable amount of time [46], which can be viewed as a ‘shortcut’ to the best up-to-date practices [44]. This result is particularly in accordance with Gabbay and LeMay’s ‘mindlines’ [44]. Indeed, these authors showed that clinicians rarely interpret clinical evidence by relying directly on scientific research, but rather rely on ‘internalized tacit guidelines’, referred to as ‘mindlines’, which are greatly reinforced by face-to-face interactions. In this approach, interactions with colleagues, but also with opinion leaders, patients and pharmaceutical representatives play a great part in the production and transfer of medical knowledge.

Our research showed a perceived gap in education and insufficient skills related to critical assessment and appraisal of scientific information, which represent an obstacle to KT. This perceived lack of evaluation skills was especially expressed by general practitioners and psychiatrists, a result which aligns with other studies showing the difficulty of evaluating information drawn from scientific medical literature, and the uncertainties concerning where to search for information to answer a specific question [46, 47].

Previous literature showed that physicians were either explicitly or implicitly expected to be aware of all available research results, in spite of an obvious lack of time and access to all medical databases [48]. Lack of time and access to information were confirmed by the participants in the study, and could figure among other barriers to KT. However, these lacks appeared more often as a suggestion for improving daily information practices. They were especially raised by general practitioners, who were the most concerned about information overload, despite the existing tools aiming at overcoming the issue such as online platforms [49].

The influence of pharmaceutical companies on publications, the organization of conferences and the choice of recommended treatments was largely assessed as a barrier to KT in our study. However, unlike other common barriers found in the literature, such as lack of time, information overload, or training gaps, dependency and biases related to pharmaceutical companies do not seem to have been raised as a barrier to KT within previous scientific literature. The publication biases raised by the surveyed physicians may be related both to self-censorship by researchers, and the rejection of negative results by publishers. Indeed, negative findings were shown to be considered of less interest than positive results in the medical literature, despite their importance in promoting and facilitating scientific communication and in preventing duplications [50].

No opposing views or disagreements were observed concerning barriers to KT during focus group discussions, or when comparing individual interviews. Instead, physicians from the five different specialties seemed to agree with the issues raised by their colleagues, and acknowledge the similarities between their practices. The only discrepancy we noted concerned the frequency of each KT barrier mentioned, depending on the medical specialty. Indeed, as mentioned above, training gaps regarding the evaluation of scientific publications were mostly reported by general practitioners and psychiatrists and less so by cardiologists and orthopaedists. Different trainings tailored to suit each medical specialist’s needs would thus be an important outcome of this study.

Using a bottom-up approach, we presented various suggestions for improvements provided by practicing physicians, leading to concrete possibilities that match the needs, concerns and preferences of physicians practicing in Switzerland regarding KT. As has been previously shown [44], using this specific approach has important implications for the dissemination of research findings. Relying on the participants’ issues, needs and suggestions transfer the locus of control from stakeholders to key field actors, leading to changes in representations and practices that are grounded in the reality of their daily practices. These research methods thus contribute to empowering field actors. Practice-based evidence [44], achieved by searching for answers by focusing on ideas emerging from clinicians themselves, seems to be a possible path for improvements in the field of KT [16].

The participants in the study suggested improving medical education, providing guidelines and journals that would be free from the influence of pharmaceutical firms, privileging cooperation between theoretical research and clinical practice, more transparency and reliability in publications, and developing several technological tools that would meet their daily needs. The physicians’ wish for synthetic, summarized, clear, local and up-to-date information aligns with previous literature stressing physicians’ needs for high-quality information, and services that would survey all sources of information and create alerts for information that is relevant and useful to their daily practice [51, 52]. The necessity of developing technological platforms and tools, such as computerized databases, search engines, and decision-support devices, has been shown and solutions have been tested aiming at helping KT for physicians and to overcome information overload in healthcare [23, 24, 53]. However, physicians’ and other healthcare practitioners’ perspectives may have not been sufficiently taken into account in the creation of those tools. Furthermore, while technological and automated management tools can be considered as supports, they cannot replace decision-making and actions by trained professionals [54] with specific, local and task-oriented needs. Physicians participating in this study have shown different levels of proficiency and awareness regarding pre-existing information and communication tools, depending on their specialty, years of practice and personal interests.

As an alternative path to the development of multiple models for translation of evidence-based information into practice from an outsider perspective, our research provides an unprecedented insider view of how physicians practicing in Switzerland deal with daily knowledge and its application into practice. This research will help to consider concrete solutions for day-to-day applications, thanks to options being suggested by the user population itself.

The question of overcoming the identified barriers is legitimate in the field of KT. The recommendations we suggest seem realistically achievable in the current Swiss context, and could be generalized to other countries. Indeed, improving training for better critical appraisal of scientific research seems achievable through continuing medical education programs. Better communication about available training may lead to an increase in participation. However, in alignment with Gabbay and LeMay [44], one idea stemming through our study would be to target the right group of people for scientific training. Indeed, a key challenge is to make sure that the people that practitioners trust, who are labelled as ‘opinion leaders’, base their knowledge on scientific evidence and can thus transmit it legitimately.

Regarding the applicability of guidelines, it is necessary to carry on needs assessment studies to develop approaches of presenting synthesized evidence more directly usable in practice. Access to literature is currently being developed in Switzerland (a national license allows free access to the Cochrane Library [55]), but further developments are needed to tailor information channels and devices to the expectations and type of activity of busy physicians. Among tools that may facilitate access to information, we could cite examples such as PEARLS (Practical Evidence About Real Life Situations) [56] or targeted short messages and easy to navigate information platforms, providing up-to-date and synthetized information matching daily physicians’ needs, and consistent with our study results.

### Study limitations

As the present study was carried out by researchers among whom were members of a medical research institution that is favourable to EBM, social desirability [57] might have influenced some interactions and opinions during interviews, FGDs and within the online survey. To counterbalance this possible bias, each moderator opened the encounters stating that they were aimed at understanding KT practices in medicine, and not at evaluating one’s medical day-to-day practice.

Another possible bias that was openly raised by a participant in a focus group is the fact that the participants received a list of all participants prior to the encounter. This list may have influenced the choice of withdrawing from the study or affected some topics of discussion. Considering that Switzerland is a small country, physicians practicing within the same specialty and in the same linguistic region may know each other and may be aware of possible conflicts of interest or preferences with their peers. However, this bias could either be negative or positive, in the sense that gathering people who know each other and may collaborate in their daily practice might have helped the fluidity of conversations, allowing physicians to discuss certain aspects of their practice openly with their colleagues. Furthermore, FGDs might have been considered as potential sharing spaces for physicians, similar to the peer networks they suggested developing in the field of possible improvements in education.

The small number of participants in the interviews and FGDs might be considered a limitation to our research, despite the fact that it aligns with qualitative method recommendations regarding the management of focus group discussions [34, 36]. The low participation rate in the online survey limits the capacity to generalize the results to Switzerland and other countries, and may reflect a lack of interest for the topic of KT, or for the type of methods used to investigate the issue. Findings obtained by content analysis of the interviews, FGDs, and open-ended questions were found to be consistent between French- and German-speaking regions. We therefore came to the conclusion that increasing the number of participants in the FGDs and interviews would not have led to very different findings and conclusions.

The inclusion criteria for study participation, namely having no hospital affiliation and having worked in a private practice for at least 5 years, may have affected our results. Indeed, results showed that some of the surveyed physicians had difficulties accessing online information, and wished for more user-friendly devices. A younger sample that would include freshly installed physicians may have led to different results, since it could be hypothesized that a younger generation of physicians may be more comfortable with computer use and more aware of the available tools. Physicians working in a hospital environment may have expressed different standpoints, too, regarding EBM and its use in daily clinical practice, thanks to their proximity to experts of different medical fields and their easier access to medical knowledge sources.

Although members of all specialties participated in the online survey and face-to-face interviews, the initial goal of performing FGDs for each specialty had to be abandoned because due to the lack of time and availability of the physicians contacted. Due to the total number of practicing physicians in Switzerland, a higher number of general practitioners participated in the online survey and in FGDs and interviews, which explains the richer data presented concerning family physicians. This research focused on discourses on practices, which involves awareness of one’s own competencies and difficulties regarding KT, and the ability to identify and put them into words for the research team. Another method would certainly have highlighted different aspects of the surveyed physicians’ practices.

Finally, across this article, an emphasis was placed on the use of a bottom-up approach to assess physicians’ needs and practices from an insider perspective. However, we recognize that the bottom-up approach was not fully achieved. Indeed, physicians who participated in the study did not contribute to the data analysis, nor did they validate what we identified as barriers and possible improvements. Study resources were limited and the participation of active physicians to the analysis would have been a paramount challenge.

Generating conversations with key stakeholders would be an important outcome of the study, since assessing their concrete suggestions could lead to changes in representations and attitudes concerning KT among physicians from various disciplines. The research team is also interested in exploring other health professionals’ views on barriers to KT and use of EBM in their daily practice. This has started to be explored by one of the authors and another researcher studying dieticians’ representations and practices of KT in Switzerland. In ongoing projects we also target the general public, directly, and as an indirect incentive to healthcare professionals to base their practice on actual evidence.

## Conclusions

The use of a multi-disciplinary and mixed methods approach enabled us to explore physicians’ preferences and needs related to KT into their daily practice. This approach provided rich, realistic and pragmatic improvement possibilities in the fields of EBM translation and implementation. Although they are prone to use EBM in their daily practice, and despite the existence of numerous models for KT as well as countless guidelines for medical practice, the surveyed physicians suggested several possible improvements, and expressed the need for more adequate and user-friendly technological tools. It is essential to create concrete possibilities for improvement in physicians’ daily practice, knowing that barriers to KT and dissatisfaction regarding available tools can generate delays in adopting effective approaches or result in an underuse of existing possibilities. Using satisfying and easy-access tools in daily practice allows the entire healthcare community to save time and energy.

## Abbreviations

EBM, evidence-based medicine; FGDs, focus group discussions; KT, knowledge translation

## Additional file

Additional file 1: Original citations from interviews, focus group discussions and online surveys. (DOC 38 kb)
