# Supplementary material for: Meeting physicians’ needs: a bottom-up approach for improving the implementation of medical knowledge into practice
Source: Health Res Policy Syst. 2016 Jul 18;14:49. doi: 10.1186/s12961-016-0120-5 (PMC4949753; doi:10.1186/s12961-016-0120-5)
Supplement: Additional file 1: — Original citations from interviews, focus group discussions and online surveys. (DOC 38 kb) [file 12961_2016_120_MOESM1_ESM.doc]

**Additional file 1**

**Original excerpts:**

***Excerpts drawn from survey question n°26:***

Excerpt 2: Online survey answer by Italian-speaking general practitioner:

"La ricerca dovrebbe essere meno dipendente dell'industria chimile. Dovrebbero essere publicate anche le ricerche di resultati negativi."

Excerpt 6: Online survey answer by French-speaking psychiatrist:

« Synergie améliorée entre recherche, clinique, et enseignement. Intégration et valorisation de la pratique et de l’expérience clinique au niveau des universités (recherche et enseignement). »

Excerpt 7: Online survey answer by German-speaking general practitioner:

"Die Guideline Autoren sollten von Interessengruppen unabhängig sein. Dann könnte man diesen eher Vertrauen Schenken. Pharmaforschung sollte auch von Staatlichen Institutionnen durchgeführt werden" "Man sollte Pharma-, apparat-, hotel-, etc industrie ganz (100%) aus der medizineschen Fortbildungen nehmen" "Empfehlungen sollen nicht primär von Industrie gesponsert sein."

Excerpt 8: Online survey answer by French-speaking general practitioner:

« Une meilleure sélection par les revues scientifiques des études afin qu’on ait moins besoin d’être suspicieux devant les résultats ».

Excerpt 13: Online survey answer by Italian-speaking psychiatrist:

"Migliorare siti scientifici su tablet"

Excerpt 14: Online survey answer by Italian-speaking general practitioner:

"Mettere un programma informatico con aggiornamento continuo"

Excerpt 17: Online survey answer by Italian-speaking cardiologist:
"Più tempo...meno pressione"

***Excerpts drawn from Focus Group discussions:***

Excerpt 3: Focus Group with French-speaking general practitioners:

« Pour moi les guidelines c’est vraiment des outils, et je trouve que de plus en plus c’est des outils qui sont très peu appropriés à notre travail. Ils sont fixés sur des mesures, qui d’abord changent tout le temps. (…) en fait on a des outils qui sont très mal calibrés pour ce qu’on fait, et quand même des fois pas très fiables. »

Excerpt 12: Focus Group with French-speaking general practitioners:

« Ce qu’il me semble en règle générale en médecine, par rapport au bombardement d’informations, c’est qu’il manque un pré-tri. Il y a pas de tri. C’est des vagues déferlantes qui nous arrivent dessus et je trouve qu’il devrait y avoir un tri. Moi je rêve par exemple qu’une fois par jour je reçoive (…) une page avec une ligne style sténographique (…) un truc super simplifié (…) il y aurait un gain d’énergie énorme (…) nous mâcher un peu le travail (…) une espèce de vrai résumé par discipline, on a pas ça, c’est un peu anarchique je trouve. »

Excerpt 15: Online survey answer by Italian-speaking orthopedic surgeon:

"Facile e gratuito accesso ai giornali"

Excerpt 16: Online survey answer by French-speaking cardiologist:

"L'accès plus large et moins coûteux aux revues médicales online"

***Excerpts drawn from face-to-face interviews:***

Excerpt 1: Interview with German-speaking cardiologist:

"Und es geht eben nicht ohne die Pharmaindustrie. Es geht nicht, wir brauchen das Geld. Wer zahlt‘s sonst, oder? Nationalfond, ist mehr so Grundlagenforschung und so weiter. Ich weiss dass wir das Geld brauchen."

Excerpt 4: Interview with German-speaking general practitioner:

"Den Background habe ich nicht, den Statistikteil einer Studie interpretieren zu können. Das ist jetzt etwas weil ich keine Wissenschaft betreibe, erstens. Zweitens, weil wir nicht hinreichend geschult worden sind in diesem Bereich. Während dem Studium und Statistiken in dem Sinn zu lesen, zu führen, zu äh wissen Sie wenn man irgendwelche Formeln hat, muss ich sagen."

Excerpt 5: Interview with German-speaking psychiatrist:

"Die Forschung ist vielleicht zu speziell, zu spezialisiert…oder vielleicht auch hat sich auch der Bezug zur Praxis ein bisschen verloren."

Excerpt 9: Interview with French-speaking diabetologist:

« Je trouve que dans la médecine c’est valable comme dans la musique : il faut bien connaître les règles pour pouvoir après s’en détacher sans faire de dégâts ».

Excerpt 10: Interview with German-speaking cardiologist:

"Das Verständnis für die vielen Fachbegriffe, das Protokoll, das ist das eine…(...) ich habe deswegen auch eine statistische, also eine kleine Ausbildung gemacht. Genau um das wirklich besser zu begreifen was das dann heisst, das relative Risiko und so weiter…ob das wirklich nach so viel tönt wies ausschaut. "

Excerpt 11: Interview with French-speaking orthopedic surgeon:

“Ce que j’ai toujours espéré (…) c’est d’impliquer les gens qui sont en cabinet dans les hôpitaux publics, que ça soit pas des gens qui sont complètement à l’écart de ce qui se passe dans un entre universitaire. C’est de faire un réseau (…) c’est plus interactif, et plus motivant pour que les gens changent un petit peu leur quotidien monotone. »

**Online Survey Question 26:**

*French*:

**Question 26:**

Quelles seraient vos suggestions pour améliorer concrètement l’application pratique des connaissances issues de la recherche ?

*Italian*:

**Domanda 26:**

Cosa suggerirebbe per milgiorare l’applicazione pratica delle conoscenze derivate dalla ricerca ?

*German*:

**Frage 26:**

Haben Sie konkrete Vorschläge, wie die Integration wissenschaftlicher Erkenntnisse in die klinische Praxis verbessert werden können?
